# Non-Surgical Management of Congenital Eversion of the Eyelids

**Published:** 2010-07

**Authors:** Caroline O Adeoti, Adeyinka O Ashaye, Michaeline A Isawumi, Ralph A Raji

**Affiliations:** 1 Department of Ophthalmology, Ladoke Akintola University of Technology (LAUTECH) Teaching Hospital, Osogbo, Osun State, Nigeria; 2 Department of Ophthalmology, University College Hospital, Ibadan, Nigeria

**Keywords:** Eyelid Diseases, Therapy, Ectropion

## Abstract

**Purpose:**

To report the effectiveness of non-invasive management of congenital eversion of the eyelids, a rare condition associated with serious socio-psychological consequences.

**Case Report:**

Three neonates with congenital eversion of the eyelids and secondary conjunctival chemosis and prolapse were managed with 5% hypertonic normal saline, lubricants, antibiotics, and padding. Complete eye opening was achieved by the 10th day of presentation and the condition resolved.

**Conclusion:**

Non-invasive management of congenital eyelid eversion was found to be effective with no need for surgical management. All health care workers should be informed that this condition is amenable to conservative treatment if started early, so that prompt referral for expert management can be offered.

## INTRODUCTION

Congenital eyelid eversion is a rare entity which usually presents at birth and more commonly involves the upper eyelid. It may be described as a condition in which the eyelid is completely turned out and is usually associated with swelling, conjunctival prolapse and chemosis. It was first reported by Adams^1^ in 1896, who used the term double congenital ectropion.

Congenital lid eversion can be due to infections, inflammation and birth trauma; or may be associated with systemic anomalies such as Down syndrome.^2^ It can also follow oculoplastic procedures for congenital ptosis.^3^ Prolonged and difficult labor has been incriminated in some cases^4^, while no known cause has been found in others.^5^ The condition is usually bilateral,^6^ but may occur unilaterally.^7^ Its incidence remains unknown as it is a very rare condition which may occur in an otherwise normal infant.^6^

Patients may be treated medically with hyperosmotic agents, antibiotics and patching; or surgically by applying temporary tarsorrhaphy using mattress sutures.^2^,^6^ Reversion of the eyelids to their normal state may be difficult in some cases due to massive inflammation and chemosis. However, the condition usually resolves within 2–3 weeks.^4^

Our first patient was encountered after twelve years of ophthalmology practice in Osun State, Nigeria and has already been reported^8^. The consequent patients and the efficacy of non-surgical management prompted our interest in this new report for which we obtained ethical approval by our institution.

The aim of the current case series is to highlight effectiveness of a noninvasive treatment approach instead of the invasive method which is still widely practiced in some parts of developing countries. We also discuss the socio-psychologic aspects of the disease, its etiopathogenesis and treatment options.

## CASE REPORTS

### Case 1

**Figure 1:**
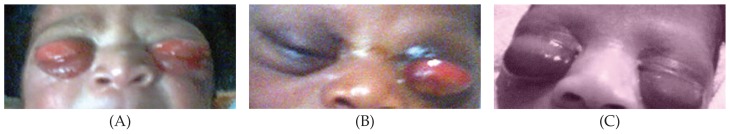
Appearance of the neonates on admission before treatment.

A 3 day-old male neonate was brought to medical attention by his parents who complained of inability to see his eyes since birth, mucopurulent discharge and excessive tearing (Fig. 1A). The baby had developed a fever 24 hours before presentation. Antenatal care had been uneventful, there was no maternal genital infection, delivery was normal vaginal and there was no report of birth trauma.

The baby was slightly febrile (temperature, 38.5°C) and without jaundice. Ocular examination revealed a purulent discharge, moderate conjunctival injection and massive chemosis in both eyes (Fig. 1A). Both upper lids were everted and the eyeball could not be seen without a speculum. No systemic abnormality was detected.

**Figure 2:**
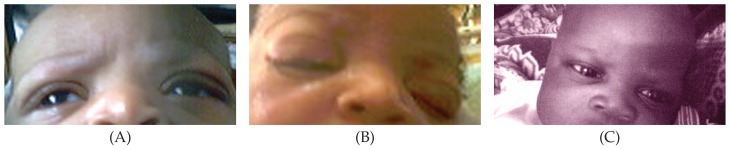
Appearnace of the same neonates as in figure 1, after treatment and on discharge.

A diagnosis of bilateral congenital lid eversion with possible neonatal sepsis was made. Pediatricians were consulted and the patient was treated as a case of neonatal sepsis with intravenous cefuroxime. The eyes were treated with tobramycin ointment, padding with 5% hypertonic saline-soaked gauze dressing and frequent application of methylcellulose gel. By the eighth day of admission, the chemosis resolved (Fig. 2A), the eyelids became well-apposed and fever subsided. Dilated funduscopy with 0.5% tropicamide eye drops revealed normal fundi in both eyes.

### Case 2

A 4 day-old boy (Fig. 1B) presented with protrusion of the left eye of 4 days’ duration. The right eye did not open spontaneously, but seemed otherwise normal. No discharge was reported. Pregnancy had been uneventful and delivery was vaginal and spontaneous. There was no history of maternal genital infection or birth trauma.

There was no history of fever or convulsions and pediatric assessment revealed no abnormalities. The left upper lid was everted with gross conjunctival injection and chemosis, but there was no discharge. Mild upper lid eversion, and mild conjunctival injection and chemosis of the right eye were also present. Both globes were otherwise normal.

The left eye was treated with tobramycin ointment, methylcellulose gel and padding with gauze dressings soaked in 5% hypertonic saline. The right eye was treated with only tobramycin ointment. The left upper lid needed to be repositioned twice. Complete resolution was achieved by the tenth day of admission (Fig. 2B). Dilated funduscopy with 0.5% tropicamide eye drops showed normal fundi.

### Case 3

A 4 hour-old male neonate was referred from a private hospital immediately after birth to the emergency room of our eye clinic with fleshy protrusion of the lids and inability to open both eyes since birth (Fig. 1C). Pregnancy had been uneventful, labor was not prolonged, and delivery was spontaneous and vaginal. The perinatal period had also been uneventful. There was no history suggestive of genital infections in the mother. There was no eye discharge, nor had any traditional eye medication been applied.

Pediatric assessment revealed no abnormality. Ocular examination revealed bilateral everted upper lids, exposed and chemotic conjunctiva covering the eyeballs and tense lids, but normal cornea and anterior chamber depths in both eyes.

The parents refused admission on financial and social grounds; he was therefore treated on an outpatient basis. Treatment consisted of daily eye cleansing, application of gauze dressings soaked with 5% hypertonic saline over the chemotic conjunctiva, chloramphenicol ointment, and padding. During follow up, he developed a mild serous discharge and ciprofloxacin eye drops were commenced every two hours. Ten days later, the baby achieved complete eye opening in both eyes (Fig. 2C). Dilated funduscopy with 0.5% tropicamide eye drops disclosed normal fundi.

## DISCUSSION

Congenital eyelid eversion is reported to be very rare; nonetheless we were confronted with 3 cases within a period of 3 years. This may confirm the reportedly higher incidence of this entity in Africans.^9^ The condition is typically bilateral, but unilateral cases have been described. One of the cases in this report was unilateral.

Several factors have been implicated in its pathophysiology including orbicularis oculi hypotonia, birth trauma, vertical shortening of the anterior lamella or vertical elongation of the posterior lamella of the eyelid with failure of the orbital septum to fuse with the levator aponeurosis, absence of an effective lateral canthal ligament and lateral elongation of the eyelid.^10^

Venous stasis during delivery may also cause marked chemosis and prolapse of the conjunctiva, leading to eversion of the eyelids.^11^ Once everted, orbicularis spasm may act as a sphincter, setting up a vicious cycle of conjunctival strangulation and edema secondary to venous stasis.^12^ The conjunctival chemosis protects the cornea from exposure and hence, corneal complications are rare.

In this report, only one patient had manifestations of neonatal sepsis. In the remaining two subjects, no condition was detected which could be attributed to the problem, as similarly reported in cases by previous authors.^5^,^6^,^13^ Labor was neither prolonged, nor difficult, and there was no evidence of maternal genital infections in any of the subjects. However, the condition could have been due to an unknown intrauterine inflammation. This could have been ascertained if the lid tissue had been examined histologically. Young^5^ in 1954, found no intrinsic defect after a complete histological examination of total lid eversion in an infant who died on the ninth day after birth. Bentsi-Enchill^14^ suggested appreciable overlapping of the lower eyelid margin by the upper eyelid as a predisposing factor. Some have proposed lid laxity as the cause of lid eversion^12^, while others believe the opposite holds true^13^.

It has been suggested that a careful history should be taken stressing on information on pregnancy and labor, and signs or symptoms of vaginal infections in the mother. A pediatrician’s assessment of the child is very important, firstly to ascertain patient health, and to rule out associated congenital anomalies or other conditions such as Down syndrome^15^ and collodion skin disease^16^. Two of three cases described herein were found to be normal, while the third had neonatal sepsis.

The role of grand multiparity and post maturity on the occurrence of this condition has been questioned by earlier work in Nigeria^13^,^17^ and is not very clear. We believe that parity of the mother and maturity of the baby may be coincidental findings rather than predisposing factors. In this report, two of the three cases were firstborns. The mode of delivery is also probably not a predisposition, as similar cases have been documented following Caesarean section.^7^ In essence, the parity, duration of labor and mode of delivery have not been shown to have a strong association.

Daily eye cleansing was performed to keep the external eye clean of any discharge, which is thought to originate from edematous lid tissues.

The mechanism by which the 5% hypertonic saline-soaked gauze dressing worked could be explained as follows: the hypertonicity allowed movement of fluid from edematous tissues through the semipermeable conjunctival membrane by the process of osmosis;^18^ this may have led to resolution of the edema and subsequent lid reversion. Two earlier reports of similar cases in Nigeria described successful early surgical management using temporary tarsorrhaphy.^13^,^17^ In another report injection of hyaluronidase into the conjunctiva before placement of lid sutures achieved resolution within 1–2 days.^19^ However, others^20^, have reported success with conservative management as we have.

The socio-psychological aspects of this condition can be serious. In Nigeria, the patient’s face is usually completely wrapped up at presentation, and naming ceremonies are usually postponed until there is significant resolution and satisfactory eye opening. By doing so, the mother protects herself from disgrace, shame and embarrassing comments.

In summary, congenital eyelid eversion can be treated conservatively. The goal of management is to prevent dessication of the exposed conjunctiva, reduction of conjunctival edema and spontaneous reversion of the lid. The patients described in this report were managed by topical lubrication, antibiotics and application of 5% hypertonic saline to the chemotic conjunctiva. We have illustrated the need for early successful repositioning of the eyelid by a conservative method rather than an invasive approach to break a cycle leading to increasing lid edema, conjunctival chemosis and eversion. A normal anatomical outcome with good cosmetic results can be effectively achieved using topical lubrication, antibiotics and application of 5% hypertonic saline. This method is highly recommended because it is inexpensive and non-invasive. It is important that all health care workers be aware of this condition so that prompt referral for expert management can be offered. Reassuring the parents should be considered of importance in the management of this condition.
